# Mapping DNA cleavage by the Type ISP restriction-modification enzymes following long-range communication between DNA sites in different orientations

**DOI:** 10.1093/nar/gkv1129

**Published:** 2015-10-26

**Authors:** Kara van Aelst, Kayarat Saikrishnan, Mark D. Szczelkun

**Affiliations:** 1 DNA–Protein Interactions Unit, School of Biochemistry, University of Bristol, Bristol BS8 1TD, UK; 2 Division of Biology, Indian Institute of Science Education and Research, Pune 411008, India

## Abstract

The prokaryotic Type ISP restriction-modification enzymes are single-chain proteins comprising an Mrr-family nuclease, a superfamily 2 helicase-like ATPase, a coupler domain, a methyltransferase, and a DNA-recognition domain. Upon recognising an unmodified DNA target site, the helicase-like domain hydrolyzes ATP to cause site release (remodeling activity) and to then drive downstream translocation consuming 1–2 ATP per base pair (motor activity). On an invading foreign DNA, double-strand breaks are introduced at random wherever two translocating enzymes form a so-called collision complex following long-range communication between a pair of target sites in inverted (head-to-head) repeat. Paradoxically, structural models for collision suggest that the nuclease domains are too far apart (>30 bp) to dimerise and produce a double-strand DNA break using just two strand-cleavage events. Here, we examined the organisation of different collision complexes and how these lead to nuclease activation. We mapped DNA cleavage when a translocating enzyme collides with a static enzyme bound to its site. By following communication between sites in both head-to-head and head-to-tail orientations, we could show that motor activity leads to activation of the nuclease domains via distant interactions of the helicase or MTase-TRD. Direct nuclease dimerization is not required. To help explain the observed cleavage patterns, we also used exonuclease footprinting to demonstrate that individual Type ISP domains can swing off the DNA. This study lends further support to a model where DNA breaks are generated by multiple random nicks due to mobility of a collision complex with an overall DNA-binding footprint of ∼30 bp.

## INTRODUCTION

The restriction-modification (RM) enzymes are important players in the prokaryotic defence against infection by foreign DNA (1–3). For the Types I, II and III RM enzymes, two main enzyme activities are directed towards the same specific DNA target sequence: a modification methyltransferase (MTase) and a restriction endonuclease. DNA target methylation protects the host genome from self-cleavage by the cognate endonuclease. Foreign DNA that has unmodified targets will be rendered harmless by the introduction of double-strand (ds) DNA breaks by the endonuclease. A striking outcome of the co-evolution of bacteria and bacteriophage over billions of years is the wide range of RM systems in the environment. Some are relatively simple - many Type II endonucleases are homodimers (4), where each monomer cuts one DNA strand, with a typical spacing of 0–4 bp between the single-strand breaks (nicks) (5). The close spatial arrangement of the active sites ensures that a dsDNA break requires just two cleavage reactions. However, a great many other RM systems have more complex structures. How these enzymes cut DNA is less clear. Here we describe experiments on the multidomain and multifunctional single polypeptide Type ISP restriction endonucleases, as exemplified by LlaGI and LlaBIII from *Lactococcus lactis* (6,7). Rather than being able to directly dimerise, structural and biochemical studies predict that Type ISP nuclease monomers are held at least 30 bp apart (8). To produce a dsDNA break it was suggested that the enzyme complex moves back-and-forth on the DNA while introducing multiple nicks, eventually shredding the DNA. Here we sought to test this model further, and to understand how the nuclease domains are activated despite not interacting directly.

The Type ISP RM enzymes recognise short, asymmetric targets; *e.g*. 5′-CTnGAYG-3′ for LlaGI or 5′-TnAGCC-3′ for LlaBIII, where the underlined thymine marks the position of the methylated adenine on the opposite strand. Since the targets are only modified on one strand (hemi-methylated), there is a potential problem for the host cell. Following semi-conservative replication, for each target copied, one of the daughter DNA will contain an unmodified sequence. This could be recognized by the endonuclease leading to self-cleavage. To avoid this, Type ISP endonucleases control their nuclease activity using long-range communication. Where there are a pair of unmodified targets in a head-to-head (HtH) orientation on foreign DNA, dsDNA breaks are produced at random, non-specific locations, with the majority between the sites (8–11). However, for every pair of HtH sites on newly-replicated host DNA, one target will retain methylation from the parental strand. This is sufficient to prevent cleavage. Although replicated host DNA will also contain pairs of unmodified sites in head-to-tail (HtT) orientation, these only support cleavage of one DNA strand (nicking) (9,10).

**Figure 1.**
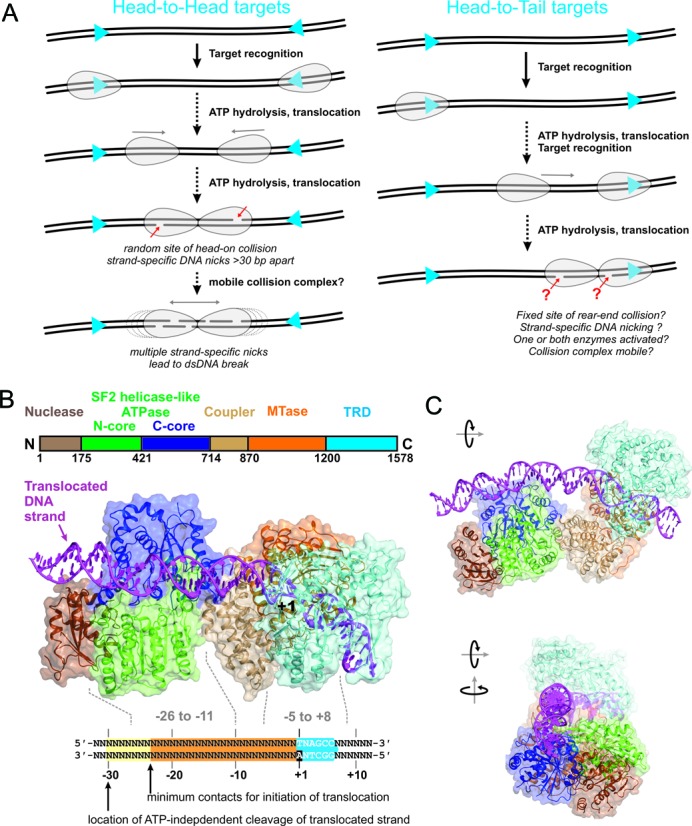
Modes of DNA cleavage and structural features of Type ISP RM enzymes. (**A**) Long-range communications and DNA cleavage by Type ISP RM enzymes on DNA with targets in HtH and HtT repeat. Targets are shown as blue arrowheads, the direction of which dictates the direction of translocation. Type ISP enzymes are shown as gray ovoids. (**B**) Model of a Type ISP restriction endonuclease–DNA complex based on PDB 4XQK with the DNA extended. DNA contacts/cleavage are defined by data from Chand *et al*. (8). DNA numbering is based on the methylated adenine (+1), with positive values in the downstream direction of translocation. The LlaBIII nucleotide sequence is highlighted as a blue box with the +1 methylated adenine in black. The orange box represents the minimum upstream contacts necessary for translocation. The yellow box represents the minimum region contacted by the nuclease. The N-terminal nuclease domain contacts the DNA at approximately -30 bp (as judged by an ATP-independent nicking event) (8). Downstream translocation occurs in a rightward direction in this view. (**C**) Rotated views of the model in panel B. Note that the nuclease is in an inactive configuration and does not contact the DNA.

The site-orientation specific communication between Type ISP targets occurs by ATP-dependent unidirectional DNA translocation (at a rate of ∼250 bp/s at 25°C) catalyzed by the helicase-like ATPase domains (10–12). Each enzyme binds independently to a target site and initiates translocation in a downstream direction (relative to the sequences shown above) (Figure 1A). DNA unwinding (i.e. true helicase activity) has not been observed. Nuclease activity is triggered wherever two translocating enzymes collide (9). Where the pair of enzymes initiate from sites in HtH orientation, the translocation is convergent and the collision complex is described as ‘head-on’. In this arrangement, each strand is cut exclusively by just one of the enzymes to give 3′ overhangs (8,11,13). Where the pair of enzymes initiate from sites in HtT orientation, collision complexes would be described as ‘rear-end’ (see below).

**Figure 2.**
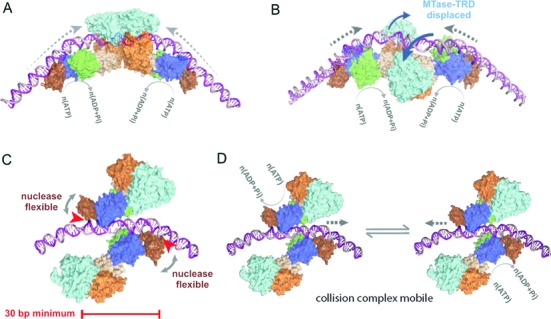
Structural models for collision and cleavage of DNA by Type ISP restriction enzymes. Structures based on PDB 4XQK (8). (**A**) Head-on collision between two translocating enzymes with the MTase-TRD domains engaged with the DNA results in placement of the nuclease domains ∼75 bp apart. (**B** and **C**) To accommodate the closer placement of the nuclease domains, the MTase-TRD domains swing off the DNA and the helicase domains collide, placing the nuclease domains at the measured ∼30 bp minimum (8). (**D**) To produce DNA nicks that are sufficiently closely-spaced to form a dsDNA break, ATP hydrolysis drives a back-and-forth mobility of the collision complex (see Figure 1A).

An X-ray crystal structure of LlaBIII bound to its target has revealed the relative disposition of the domains (Figure 1B and C) (8). Surprisingly, both the ATPase and nuclease domains are upstream of the TRD and MTase. If the enzymes in a head-on collision complex retained the same relative domain placements, this would locate the nuclease domains ∼75 bp apart (Figure 2A) (8). When we mapped the strand breaks for single dsDNA cleavage events, we found that the distance between the 3′ nicks was variable and surprisingly wide. The distances ranged from a minimum of 30 bp to >120 bp, with a median of 50–60 bp (8). We proposed a DNA cleavage model were the initial collision complex only nicks the DNA. Continued ATP hydrolysis then allows the complex to move backward-and-forward while the nuclease domains continue to nick the DNA (Figure 2B–D). Thus, nicks accumulate around the location of the initial collision and eventually result in a dsDNA break (Figure 1A). Consistent with this model, the distance between the outermost nicks widened with reaction time (8).

We interpreted the minimum 30 bp spacing observed as resulting from the closest approach of two nuclease domains within an initial collision complex. This can be accommodated by the structure if we allow the MTase-TRD domains to swing off the DNA (Figure 2B and C). The minimal DNA-binding footprint for each enzyme in a symmetrical head-on collision complex would therefore be ∼15 bp. However, direct interaction of the nuclease domains is still not possible. We therefore predict that long-range allosteric activation of the nuclease domains must occur upon collision. The underlying mechanism for this is currently unclear. For head-on collisions, the interaction interface may be the MTase-TRDs domains (e.g. Figure 2A) or the helicase domains (e.g. Figure 2C). Because of the wide range of cleaved ends observed, it is also possible that multiple different conformations of head-on collision complex exist, all of which can lead to activation of the nuclease domains. Further flexibility in the activation mechanism is evinced by the DNA nicking produced following communication between HtT targets. It has been proposed that rear-end collision between a translocating enzyme and an enzyme at its site leads to nuclease activation (Figure 1A), a quite different arrangement to head-on collision. The co-directional orientation of the enzymes means that the nuclease domains are both targeted to the same DNA strand, hence only producing nicks (9,10). However, the location(s) of the nicking events has not been mapped for HtT sites, and it has not been determined whether one or both enzymes are activated upon rear-end collision.

A complication of our previous cleavage mapping was that collision occurred at random locations following both stochastic DNA translocation events and variable conformational rearrangements of the collision complex (8). Cleavage positions were further influenced by the preference of the Type ISP Mrr nuclease for certain dinucleotide sequences. To try to simplify the analysis here, we mapped cleavage events following collision between a translocating enzyme and a static enzyme bound to its site. This approach offers the advantage that the DNA sequence in the locality of collision is invariant for each collision event. In addition to re-visiting head-on collision, we could, for the first time, map how rear-end collision on HtT substrates results in DNA nicking (9,10). This offers a view of nuclease activation in an alternative collision configuration. Head-on and rear-end collisions at a site are not necessarily rare events in a cell (14). Both may occur whenever a translocating enzyme searching for a partner reaches an enzyme bound to its site which has yet to initiate translocation. Rear-end collision complexes are particularly significant on the host DNA since, following replication, each daughter DNA will have pairs of HtT sites where both targets are unmodified.

## MATERIALS AND METHODS

### Proteins and DNA

Wild type and mutated LlaGI and LlaBIII were purified as described previously (9,10). All other enzymes were obtained from New England Biolabs or Affymetrix.

**Figure 3.**
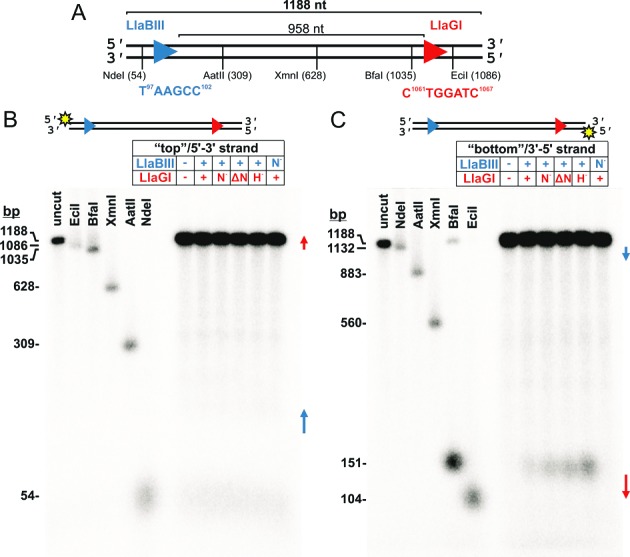
Cleavage of head-to-tail DNA results from rear-end collision between a translocating enzyme and an enzyme at a site. (**A**) Cartoon of the linear DNA substrate. The arrows show the orientations of the LlaBIII (blue) and LlaGI (red) targets, where the arrowheads indicate the direction of translocation (viz. Figure 1A). Type II restriction enzyme positions are given for the nucleotide 5′ to the phosphodiester cleaved on the top strand (according to standard definitions (5)). DNA (5 nM), 5′-labeled on either the top strand (**B**) or bottom strand (**C**) with 32-phosphorus (sun symbols), was incubated with the enzymes shown (200 nM of each) for 2 min. ATP was added to 4 mM, the reaction incubated for 10 min at 25°C and then stopped. Products were separated by alkaline denaturing agarose gel electrophoresis. The size of each marker represents the length of the labeled ssDNA produced. Table key: No enzyme (−); WT enzyme (+); nuclease point mutant (*N*−); nuclease domain deletion (Δ*N*); or, ATPase point mutant (*H−*). Gels shown are representative examples from two repeat reactions.

**Figure 4.**
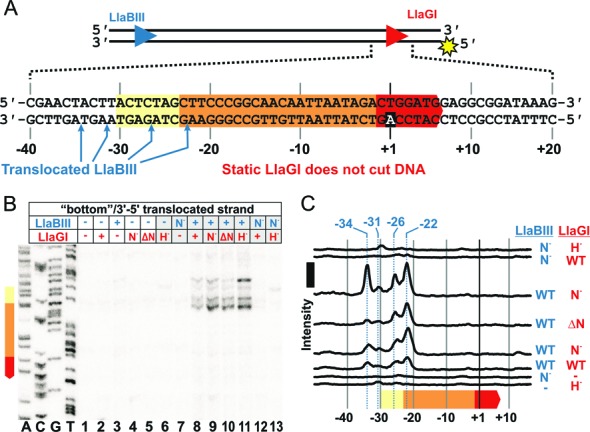
Mapping the cleavage events on the bottom strand following rear-end collision at a site. (**A**) Cartoon of the linear DNA substrate from Figure 3. The sequence of the LlaGI site is highlighted, along with the locations cleaved by LlaBIII. The sun symbol shows the labeling with 32-phosphorus. (**B**) DNA (2 nM), 5′-labeled on the bottom strand with 32-phosphorus (sun symbols), was incubated with the enzymes shown (200 nM of each) for 2 min. ATP was added to 4 mM, the reaction incubated for 10 min at 25°C, and then stopped. The products were separated by denaturing polyacrylamide gel electrophoresis. (**C**) Quantified band intensities for lanes 6–13 (in grey) in panel B (Materials and Methods). Bar represents 500 intensity units. Tables key: No enzyme (−); WT enzyme (+ or WT); nuclease point mutant (*N−*); nuclease domain deletion (Δ*N*); or, ATPase point mutant (*H−*). Gel and quantitation shown are representative examples from two repeat reactions.

**Figure 5.**
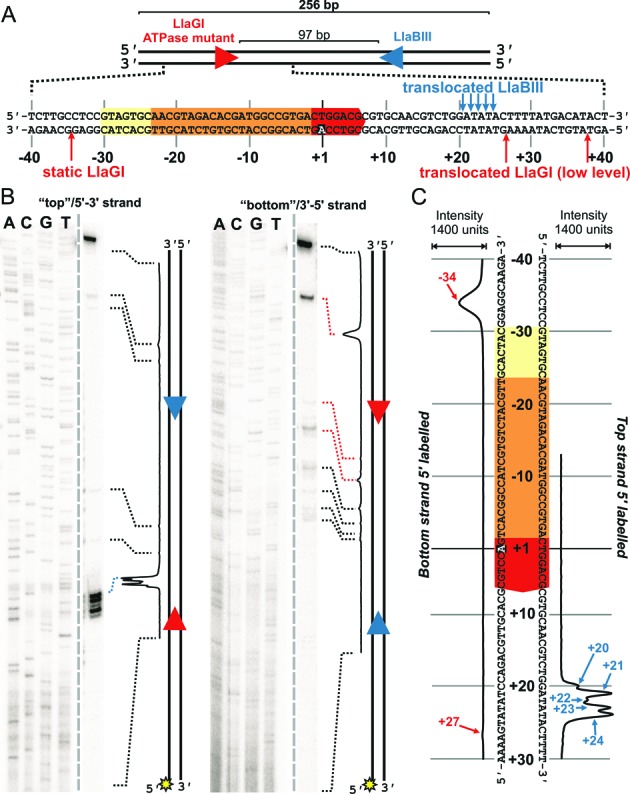
Mapping the cleavage events following head-on collision at a site. (**A**) Cartoon of the linear DNA substrate. The sequence around the LlaGI site is highlighted, along with the locations cleaved by LlaGI and LlaBIII. Some of the LlaGI cleavages are low level events due to escape of the ATPase mutant from the site (see main text). (**B**) DNA (2 nM), 5′-labeled on either top or bottom strand with 32-phosphorus (sun symbols), was incubated with the wild type LlaBIII and a LlaGI Walker A ATPase mutant (200 nM of each) for 2 min. ATP was added to 4 mM, the reaction incubated for 5 minutes at 25°C, and then the reactions stopped. The products were separated by denaturing polyacrylamide gel electrophoresis. The gray dashed line indicates where lanes have been removed (see Supplementary information for the complete gel). The dotted lines show where DNA bands align to peaks in the quantitation; those coloured in blue or red are shown in panel (A) and/or (C). (**C**) Quantified data from panel B shown in relation to the sequences up- and downstream of the LlaGI site. Gel and quantitation shown are representative examples from two repeat reactions.

The linear DNA for the mapping and footprinting experiments were generated by PCR, with either the forward or reverse primer being ^32^P-labeled at the 5′ end using T4 polynucleotide kinase and γ^32^P-ATP by standard techniques (15). The 1188 bp linear HtT mixed DNA (Figures 3 and 4) was generated from the 1739-240 region of pUC19 (16) using primers KA084F (5′-CTGGCCCCAGTGCTGCAATGATAC-3′) and KA084R (5′-GGCGCCTGATGCGGTATTTTCTC-3′). The 256 bp linear HtH mixed DNA (Figure 5) was generated from the 6–261 region of pEX-A-KA1 (see below) using primers F80 (5′-CACGATGAAGAACTATCTGCTTCCGATTGTG-3′) and R80 (5′-ATTATGGGTTTGTTGCACGGGTTGGTC-3′).

**Figure 6.**
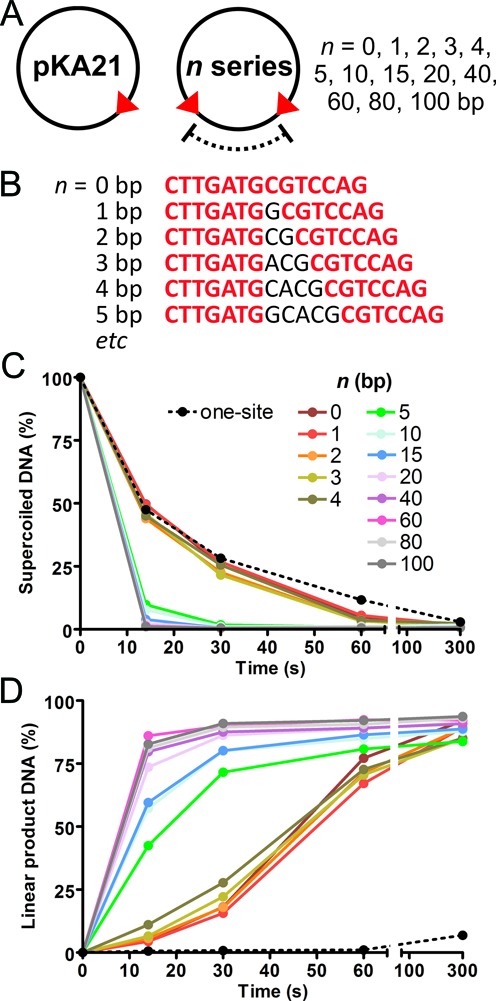
Effect of inter-site distance on cleavage of head-to-head substrates. (**A**) Plasmid substrates used in the assays. Arrowheads denote the position and orientation of the 6 bp LlaGI target sequence. (**B**) Example of LlaGI sequences for *n* = 0 to 5 bp. Plasmid DNA (2 nM) was pre-incubated with 200 nM LlaGI for 2 min at 25°C. Cleavage was initiated by addition of ATP (to 4 mM). Samples were taken at the times indicated, stopped, and analyzed by agarose gel electrophoresis. The graphs show the amount of supercoiled substrate DNA remaining (**C**) and linear DNA produced (**D**) as a function of time following addition of ATP. Points are the average of two repeat experiments.

The one-site LlaGI plasmid pKA21 was described previously (8). To generate the *n*-series DNA substrates (Figure 6), reverse PCR was carried out with the HtH LlaGI substrate pKA20 (8), using primer ROli LlaGI Int0 (5′-CATCAAGGTCTGCCTTGTATTCCCTCGCCCAC-3′) and the following partner primers: 5′-CGTCCAGTCACGGCCATCGTGTCTACGTTG-3′ (FOli LlaGI Int0, *n* = 0 bp, pKA20.0); 5′-GCGTCCAGTCACGGCCATCGTGTCTAC-3′ (FOli LlaGI Int+1, *n* = 1 bp, pKA20.1); 5′-CGCGTCCAGTCACGGCCATCGTGTCTAC-3′ (FOli LlaGI Int+2, *n* = 2 bp, pKA20.2); 5′-ACGCGTCCAGTCACGGCCATCGTGTCTAC-3′ (FOli LlaGI Int+3, *n* = 3 bp, pKA20.3); 5′- CACGCGTCCAGTCACGGCCATCGTGTCTAC-3′ (FOli LlaGI Int+4, *n* = 4 bp, pKA20.4); 5′-GCACGCGTCCAGTCACGGCCATCGTGTC-3′ (FOli LlaGI Int+5, *n* = 5 bp, pKA20.5); 5′-ACGTTGCACGCGTCCAGTCACGGCCATC-3′ (FOli LlaGI Int+10, *n* = 10 bp, pKA20.10); 5′-TCCAGACGTTGCACGCGTCCAGTCACGG-3′ (FOli LlaGI Int+15, *n* = 15 bp, pKA20.15); 5′-GTATATCCAGACGTTGCACGCGTCCAGTCACGGC-3′ (FOli LlaGI Int+20, *n* = 20 bp, pKA20.20); 5′-CACTGAAGTATGTCATAAAAGTATATCCAGACGTTGCAC-3′ (FOli LlaGI Int+40, *n* = 40 bp, pKA20.40); 5′-AACTTCAACCACAACATCTGCACTGAAGTATGTC-3′ (FOli LlaGI Int+60, *n* = 60 bp, pKA20.60); 5′-ATATGATGCAGCTTAAGGTGAACTTCAACCACAACATCTG-3′ (FOli LlaGI Int+80, *n* = 80 bp, pKA20.80); and 5′-CAAGCAATGCGGATTCTTCAATATGATGCAGCTTAAG-3′ (FOli LlaGI Int+100, *n* = 100 bp, pKA20.100v2). [Note that pKA20.100v2 has exactly 100 bp between the LlaGI sites whereas pKA20.100 (8) has 97 bp between the sites]. The PCR reactions were digested with DpnI, the linear product purified from an agarose gel and recircularized using T4 DNA ligase, and the inter-site spacing checked by DNA sequencing (www.dnaseq.co.uk). Plasmid DNA was prepared and ^3^H-labeled as described previously (17), except that the DNA was purified using a Qiagen midiprep kit. To generate the mixed HtH plasmid pEX-A-KA1, a 266 bp insert was gene synthesized and cloned in the MCS of pEX-A by Eurofins MWG Operon. The insert was based on the 1215–1481 region of pKA20.100 except that the LlaGI site at 1293–1299 was replaced by the LlaBIII recognition sequence, with the same orientation.

**Figure 7.**
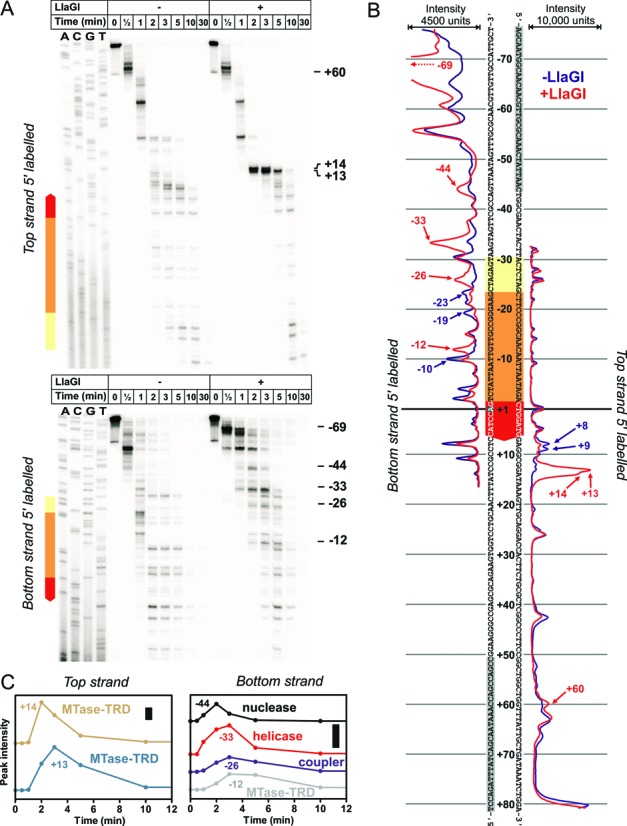
Exonuclease III DNA footprinting of a wild type LlaGI-DNA complex in the absence of ATP. (**A**) DNA (2 nM), 5′-labeled on the top strand (*upper gel*) or bottom strand (*lower gel*) with 32-phosphorus was pre-incubated without (−) or with (+) 200 nM LlaGI in the absence of ATP for 2 min. Exonuclease III (to 0.75 U/μl) was added at 25°C and reacted for the times shown. Quenched products were separated by denaturing polyacrylamide gel electrophoresis. (**B**) Band intensity summated from all time points in panel A (see Materials and Methods and main text for further details). The complete sequence of the 157 bp linear one-site LlaGI DNA is shown, with the PCR primers highlighted in gray. (**C**) Peak heights for the bands indicated as a function of exonuclease III incubation time. Bars represent 1000 intensity units. Positions are assigned to domains as explained in Figure 8 and the main text. Gels and quantitation shown are representative examples from two repeat reactions.

The 157 bp linear one-site LlaGI DNA (Figure 7) was generated from the 1785–1941 region of pUC19 using primers FOli LlaGI +75 (5′-TCCAGATTTATCAGCAATAAACCAGCCAGCC-3′) and ROli LlaGI -75 (5′-AGCAATGGCAACAACGTTGCGCAAACTATTAAC-3′).

### Mapping DNA cleavage on mixed substrates

Linear DNA ^32^P-labeled on one or other strand at the 5′ end (2 nM) was pre-incubated with 200 nM of each enzyme (as indicated) for 2 minutes at 25°C in TMDK buffer [50 mM Tris–Cl, pH 8.0, 10 mM MgCl_2_, 150 mM KCl, 1 mM DTT]. Reactions were initiated by addition of ATP (to 4 mM) and incubated at 25°C for either 10 min (for HtT mixed DNA) or 5 min (for HtH mixed DNA).

For alkaline denaturing 2% (w/v) agarose gel electrophoresis in 50 mM NaOH, 1 mM EDTA, samples were stopped with 0.5 volumes of Alkaline Buffer [300 mM NaOH, 6 mM EDTA, 18% (w/v) Ficoll 400, 0.1% (w/v) Bromocresol green and 0.1% (w/v) Xylene Cyanol]. Markers were generated by cleaving the labeled DNA with the Type II restriction enzymes shown, according to the supplier's recommendations. Following electrophoresis, the gels were neutralized for 1 hour in 500 mM Tris–HCl (pH 8), compressed for 1 h and dried under vacuum for 1 h. The dried gels were exposed to a storage phosphor screen (Fujifilm) and scanned using a Typhoon phosphorimager (GE). The 16-bit densitometric scans were analyzed using the 1D gel analysis software of ImageQuant (GE).

For urea-denaturing 8% (w/v) polyacrylamide sequencing gel electrophoresis in 1× TBE, samples were stopped with 0.5–2 volumes of STOP Buffer [80% (v/v) formamide, 10 mM EDTA, 1 mM NaOH, 0.1% (w/v) Bromophenol blue], and heated for at least 2 min at 95°C. Sequencing lane markers were produced from parent plasmids using the Sequenase Version 2.0 DNA Sequencing Kit (Affymetrix) and ^32^P-labeled primers specific for the top or bottom strands. Gels were pre-run for ∼ 1 h and the samples separated at a fixed power (80 W) for ∼1 h. Gels were dried under vacuum for 1 h. Dried gels were exposed to a storage phosphor screen (Fujifilm), which was scanned using a Typhoon phosphorimager (GE). The 16-bit densitometric scans were analyzed using the 1D gel analysis software of ImageQuant (GE). Lane pixel positions were converted to DNA lengths/positions by comparison to the sequencing lanes according to Šišáková *et al*. (10).

### Plasmid DNA cleavage assay

Plasmid DNA (2 nM) was pre-incubated with 200 nM LlaGI for 2 min at 25°C in TMD buffer [50 mM Tris–Cl, pH 8.0, 10 mM MgCl_2_, 1 mM DTT]. Reactions were initiated by addition of ATP (to 4 mM) and incubated at 25°C for the times indicated. Reactions were stopped with 0.5 volumes of 3 x STEB [0.1 M Tris (pH 7.5), 0.2 M EDTA, 40% (w/v) sucrose, 0.4 mg/ml bromophenol blue], and analyzed by agarose gel electrophoresis. The percentage of ^3^H-labeled DNA in each band per lane was ascertained by scintillation counting (17).

### Exonuclease III footprinting

Linear one-site DNA (2 nM) was pre-incubated with 200 nM LlaGI for 2 min at 25°C in TMD buffer. Exonuclease III was added to a final concentration of 0.75 U/μl and the reaction quenched at the times indicated by addition of 3 volumes of STOP buffer. Samples were separated by denaturing polyacrylamide gel electrophoresis alongside sequencing markers, and analyzed, as above.

Since cleavage of DNA by exonuclease III is stochastic and varies with sequence, even in the absence of protein roadblocks a discrete pattern of bands is observed which represent sequences where the rate of exonuclease III progression is slowest (18). Hence, the effect of a protein roadblock is best ascertained by comparing the kinetics of exonuclease III cleavage with and without the protein roadblock. Here we summated the band intensities at each time point, which allows roadblock-specific pausing of exonuclease III to be more easily identified. However, because we used the same finite set of time points for experiments with or without LlaGI, there is a sampling effect that occurs downstream of exonuclease III pause sites produced by LlaGI. This occurs because any rate-limiting step followed by a faster step in a kinetic series produces an apparent under-occupancy of the downstream species when compared to a series where all rates are the same (19). The analysis problem can be overcome by collecting an infinite number of time points (in practical terms, having a method to monitor each DNA position in real time). However, due to practical limitations, we can only analyze a finite number of samples at fixed time points. As the impedance of exonuclease III by the LlaGI roadblock(s) is greater than that produced by DNA sequences, this sampling effect means that sequences immediately downstream can appear under-occupied in the presence of LlaGI (Figure 7).

## RESULTS

### Nicking of DNA with HtT targets is due to activation of the motile enzyme following rear-end collision with an enzyme at a site

We first sought to understand how DNA nicking occurs during long-range communication between sites in HtT orientation (9,10). We wanted to know where the nicking occurs and which enzyme(s) in the collision complex are catalyzing the reaction. To examine this we utilized mixed HtT DNA substrates with one LlaBIII site and one LlaGI site (Figures 3A and 4A). The amino acid sequence of LlaBIII and LlaGI are almost completely identical outside of the TRDs and the enzymes can cooperate to cleave mixed DNA substrates (10,11). The advantage of using the mixed DNA is that because LlaBIII and LlaGI recognise unique targets, we can mix one wild type (WT) enzyme with a second protein carrying a nuclease point mutation (*N−*), a nuclease domain deletion (Δ*N*, where the nuclease domain residues 1–165 is deleted) or an ATPase point mutation (*H−*), or any combination thereof. The DNA substrate was generated by PCR, so that we could label either the top (5′-3′) or bottom (3′-5′) strand with 32-phosphorus (Materials and Methods). Elevated concentrations of the Type ISP enzymes were used (200 nM) as these conditions are necessary to produce DNA cleavage *in vitro*: the same concentrations were used previously when mapping cleavage produced by head-on collision between translocating enzymes (8–10).

We first examined the nicking of the HtT mixed DNA (Figure 3A) using alkaline denaturing agarose gel electrophoresis (Figure 3B and C). This technique allowed us to observe cleavage at any point along the labeled strand both up- and downstream of the sites (Materials and Methods) (11). Enzyme-dependent cleavage of the top strand was not observed using any enzyme combinations. Instead, cleavage occurred on the bottom strand, exclusively at a region upstream of the LlaGI site (Figure 3B and C). This is consistent with a model for collision-dependent activation at a site (Figure 1A), where the translocating enzyme is LlaBIII and the static enzyme bound at the site is LlaGI. DNA nicking was still observed when WT LlaGI was exchanged for either a nuclease mutant, a nuclease domain deletion mutant or a Walker A ATPase mutant, but was not observed when WT LlaBIII was exchanged for its nuclease mutant (Figure 3C). This suggests that, following rear-end collision, the translocated LlaBIII is activated to cut one of the DNA strands while the static LlaGI does not cut the DNA at all.

Relative to WT LlaGI, the LlaBIII nicking activity was enhanced by collision with each of the LlaGI mutants (Figure 3C). For the ATPase mutant this is most likely because translocation cannot initiate. This would lead to a longer lived roadblock. We show below that this enhancement is not due to activation of the nuclease domains of the LlaGI ATPase mutant. For the nuclease mutants the enhancement may be due to increased stalling during initiation which also leads to a longer lived roadblock. Similar stalled states were observed with nuclease mutants of other Type I enzymes (20).

To pinpoint the exact DNA locations cleaved by LlaBIII, we ran the bottom strand samples on a denaturing polyacrylamide sequencing gel that allows for single nucleotide resolution (Figure 4B,4C) (Materials and Methods). Four distinct cleavage loci were observed upstream of the LlaGI site (at positions −34, −31, −26 and −22, with −34 and −22 being the principal products). As above, the cleavage products were not observed when WT LlaBIII was exchanged with its nuclease mutant (lanes 7 and 12), confirming that all the products resulted from LlaBIII activity and were not due to activation of LlaGI. For reactions with the Walker A ATPase mutant of LlaGI, the enhanced cleavage seen in lane 11 was not the result of catalysis by the LlaGI mutant; when the WT LlaBIII was exchanged for its nuclease mutant, very little cleavage was observed except for a weak non-specific cleavage at −31 (lane 13), which was also observed with the LlaGI ATPase mutant alone (lane 6). We did not see any cleavage downstream of −22 with any combination of enzymes. Once LlaGI starts translocating or once the LlaGI MTase-TRD is displaced completely from the DNA, either the LlaBIII nuclease becomes deactivated or the complete enzyme dissociates from the DNA. A model for how LlaBIII produces multiple cleavages is discussed below.

### Head-on collision at a site results in nuclease activation of both enzymes

To investigate head-on collision at a fixed location, we examined DNA cleavage on a mixed substrate with HtH sites (Figure 5A) using WT LlaBIII and a LlaGI Walker A ATPase mutant. This combination ensures that the majority of the collisions occur between a translocating molecule of LlaBIII and a static molecule of LlaGI bound to its site. The HtH DNA was labeled with 32-phosphorus on one or other strand, incubated with the enzymes for 5 min and the cleavage products separated on a denaturing polyacrylamide sequencing gel (Figure 5B) (Materials and Methods). In contrast to the HtT DNA, cleavage of both strands was observed, but the minimum distance between the nick sites was too distant (55–59 bp) to produce efficiently a double-strand break (see Figure 6B in (11)). The cleavage loci were assigned to either LlaBIII or LlaGI on the basis of strand specificity of cleavage (Figure 5A and C). We have shown previously (11), that in a head-on collision, the leftward enzyme (LlaGI here) cuts exclusively at the bottom strand while the rightward enzyme (LlaBIII here) cuts exclusively at the top strand. Therefore, the cleavage at -34 is due to activation of the LlaGI nuclease while the cleavages at +20 to +24 are due to activation of the LlaBIII nuclease. Cleavage events were observed at other locations on the bottom strand with a reduced frequency (marked by the dotted lines in Figure 5B). This suggested that the LlaGI ATPase mutant had translocated away from its site to some extent. A low amount of cleavage on the top strand upstream of the LlaBIII site was also observed (marked by the dotted lines), which may have resulted from collision with the weakly-translocating LlaGI ATPase mutant, or from an ATP hydrolysis- and collision-independent nuclease activity of LlaBIII (8).

The multiple LlaBIII cleavages on the top strand (Figure 5) may represent favourable nucleotide sequences for cleavage, domain flexibility and/or multiple collision complexes with different protein-protein interfaces (see Discussion). We note that the +20 to +24 region of the DNA used here is particularly AT rich and these dinucleotides are cleaved with an elevated frequency (8).

Although the nuclease domain of the LlaGI ATPase mutant was activated following head-on collision by the translocating LlaBIII, cleavage was observed at principally just one location (Figure 5B and C). We note that wild type LlaGI in the absence of ATP also cut its upstream DNA at one location (−30 in Figure 1B), albeit at a much slower rate (8). The difference in cleavage position here may be due to local sequence variation between the DNA substrates used or may reflect a structural difference in the ATPase mutant. In both cases, cleavage at a single location in the absence of ATP hydrolysis may be because the nuclease domain position is fixed; remodeling of the enzyme complex driven by ATP hydrolysis may allow cleavage at multiple locations due to increased nuclease domain flexibility or to movement of the whole Type ISP enzyme.

### Evaluating the extent of downstream DNA bound by LlaGI

To help interpret the data generated above on the HtH DNA, and in particular to help define the location of the collision interface, we needed to confirm how much downstream DNA is encompassed by LlaGI when bound to its site. To evaluate this we measured the rate of DNA cleavage by LlaGI on HtH plasmids where the inter-site distance was varied between 0 and 100 bp (Figure 6A,6B) (Materials and Methods). Where the inter-site distance becomes too short to accommodate two Type ISP enzymes bound at the same time, dsDNA cleavage will become inhibited. DNA cleavage was compared to a one site DNA which is at best nicked due to rear-end collisions following translocation of the entire circular DNA (9,10).

The disappearance of supercoiled DNA substrate measures the rate of the first strand cleavage event. For DNA with an inter-site distance of 0–4 bp, the initial cleavage rate was similar to that of a one-site DNA (Figure 6C). This suggests that two enzymes cannot bind the sites simultaneously at this site-spacing. For inter-site distances of 5 bp or greater, the initial cleavage rate was more rapid and within error invariant. This faster rate is characteristic of communication of two enzymes between the HtH sites, and suggests that there is sufficient downstream DNA for saturated binding of each site by one enzyme. In the crystal structure, the MTase-TRD encloses the DNA as far as position +8 (Figure 1B and C) (8). The specific target sequence is from +1 to +6, so 2 bp downstream of the site are contacted. Therefore, one might predict that the minimum spacing between two HtH targets that would allow simultaneous binding would be at least 4 bp. Therefore, there is a close correspondence between the crystal structure and the kinetic data.

The formation of linear DNA product measures the rate of the final nicking event that generates a dsDNA break (Figure 6D). The one-site DNA does not generate linear product as all DNA cleavage is targeted to one strand (*viz* Figure 3). The DNA with inter-site distances of 0–4 bp generated linear DNA more slowly than HtH DNA with longer spacings, at a rate similar to the initial nicking event. We suggest that double-strand cleavage of these DNA results from translocation of one enzyme around the entire plasmid and collision with a second enzyme that binds in the meantime at the opposite site. Such events would have a reduced probability which would limit the rate. Moreover, additional rate-limiting remodeling events may be required before two nicks are sufficiently close to generate a dsDNA break. For the DNA with an inter-site distance of 5 bp, the rate was noticeably faster than the 4 bp spacing (Figure 6D). We suggest this is because two enzymes can now associate at the same time allowing head-on interaction without translocation around the entire plasmid. We note that the dsDNA cleavage rate continued to increase with inter-site distance, saturating at 20–40 bp (Figure 6D). This increase may reflect that at inter-site distances of 5–20 bp, the majority of the cleavage events resulted from collision between an initiated translocating enzyme and an enzyme still bound to its site prior to initiation (14). The remodeling events necessary to generate a dsDNA break may limit the observed rate.

### Exonuclease footprinting reveals domain boundaries for LlaGI bound to its site

To explore further the positioning and flexibility of the Type ISP domains when bound at a site prior to ATP hydrolysis, we examined the outer limits of the LlaGI–DNA contacts in the absence of ATP using DNA footprinting with exonuclease III from *Escherichia coli*, which has 3′→5′ exonuclease activity (Figure 7) (Materials and Methods). The DNA substrate was a PCR product with a single, centrally-located LlaGI site (the complete sequence of the PCR product is shown in Figure 7B). The DNA was labeled on either top or bottom strand with 32-phopsphorus, incubated with or without WT LlaGI, and then the progress of exonuclease III digestion from the 3′ ends monitored at different time points by separation of the cleavage products on denaturing polyacrylamide gels (Figure 7A) (Materials and Methods). Note that the 3′→5′ progress of exonuclease III is stochastic and the rate of phosphodiester cleavage depends on local sequence (C>A = T>G) (18). Consequently, a banding pattern is observed even in the absence of LlaGI (Figure 7A). To determine locations where LlaGI acts as a roadblock to exonuclease III progression, each lane was scanned and the intensity of the lanes summed. By comparing the summed intensities we can identify protein interaction as either enhanced cleavage by exonuclease III (due to the roadblock effect increasing the exonuclease lifetime) or reduced cleavage by exonuclease III (the latter either because LlaGI binding changes the sequence preference of exonuclease III or because of a sampling effect which is described in more detail in Materials and Methods).

**Figure 8.**
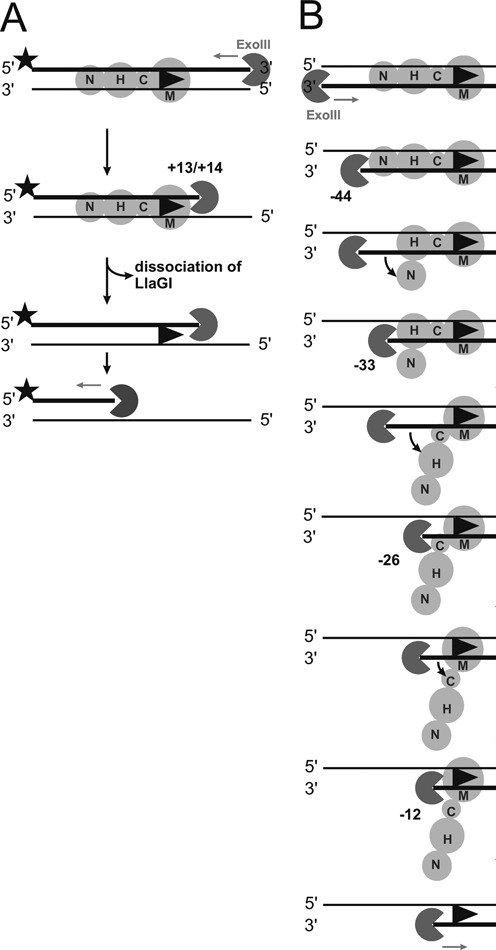
Models to explain the footprinting data in Figure 7. (**A**) Model for products produced during 3′→5′ digestion of top strand-labeled DNA by exonuclease III. (**B**) Model for products produced during 3′→5′ digestion of bottom strand-labeled DNA by exonuclease III. In both panels, exonuclease III is shown as a Pac-Man, the 6 bp target site as an arrowhead, labeling with 32-phosphorus by the star symbol, and LlaGI as a series of circles representing the domains: nuclease (N), helicase (H), coupler (C) and MTase-TRD (M).

Using DNA 5′-labeled on the top strand (Figure 7A *upper gel*), we were able to follow the approach of exonuclease III towards LlaGI on the downstream side (i.e. as it digested the top strand starting at the 3′ end and moved towards the MTase-TRD domains). The summated scan data in Figure 7B reveals a clear enhancement at positions +14 and +13. These most likely result from the collision between the exonuclease III and the MTase-TRD bound to the site (Figure 8A), with the reduced cleavage at +8 and +9 due to the sampling effect when LlaGI releases the site. Note that because of the DNA binding envelope of exonuclease III and location of its active site relative to the leading edge of the protein (21), the observed exonuclease III cleavage locations are an upper bound for the position of a roadblock. i.e., the actual edge of the LlaGI structure is not at +13/+14 (viz. Figure 1B). Importantly, no further enhancements/reductions were observed on the top strand upstream of +8, suggesting that progression of exonuclease III occurs due to the dissociation of the complete LlaGI enzyme from the DNA. Were the helicase/nuclease domains to remain bound, we would have expected to observe additional enhanced cleavages upstream of +13. An additional more minor enhancement at +60 was also observed, which may represent some interaction of LlaGI near DNA ends (similar to the bottom strand, see below).

Using DNA 5′-labeled on the bottom strand (Figure 7A *lower gel*), we were able to follow the approach of exonuclease III towards LlaGI on the upstream side (i.e. as it digested the bottom strand starting at the 3′ end and moved towards the nuclease-ATPase domains). The summated scanned data in Figure 7B reveals a number of clear, stepwise changes that we suggest represent discrete roadblocks to exonuclease III progression produced by different LlaGI domains, namely: the nuclease (enhancement at −44), the helicase (enhancement at −33), the coupler (enhancement at −26) and the MTase-TRD (enhancement at −12) (Figure 8B). The reduced cleavage events at −23, −19 and −10 most likely represent sampling events due to the enhancements at −26 and −12 (Materials and Methods). It could be argued that an alternative source of the enhancement at −33 is an ATP-independent cleavage event by LlaGI (8). However, such cleavage was previously only observed after ∼1 hour incubation whereas the footprinting band at −33 appeared within ∼2 min (Figure 7A). The LlaGI nuclease is also unlikely to be activated by the rear-end collision of exonuclease III as collision in this orientation does not activate the Type ISP nuclease (viz. Figures 3 and 4). The additional enhancement observed at -69 may represent an end binding effect as seen on the top strand.

The band intensities at each time point at locations +14 and +13 on the top strand and at −44, −33, −26 and −12 on the bottom strand are plotted in Figure 7C. The upward gradient represents the appearance of that band on the gel, which in turn represents the times at which exonuclease III arrived at the roadblock. The downward gradient represents the disappearance of the band on the gel, which in turn represents the times when exonuclease III progressed, dependent upon the DNA-binding lifetime of the roadblock (18). On the upstream side (bottom strand labeled with 32-phosphorus), the data are consistent with the stepwise arrival at, and subsequent displacement of (Figure 8B): firstly, the nuclease domain (−44); secondly, the ATPase domain (−33); and finally, the coupler-MTase-TRD (−26 and −12). The dissociation of the nuclease and ATPase domains was >3-fold faster than the dissociation of the coupler-MTase-TRD. The higher stability of the coupler-MTase-TRD is corroborated by the time course of the +14/+15 positions on the downstream side (top strand labeled with 32-phosphorus), which show a similar dissociation kinetics (Figure 7C).

The footprinting data therefore shows that the domains of LlaGI can, to some extent, separately dissociate from the DNA, and that the interactions of the nuclease and helicase are less stable that those of the coupler-MTase-TRD in absence of ATP.

## DISCUSSION

Using a combination of DNA cleavage site mapping by gel electrophoresis, kinetic analysis of DNA cleavage and exonuclease III footprinting of DNA-binding, we have been able to shed more light on how the multiple domains of the single polypeptide Type ISP RM enzymes can interact to activate nuclease activity. A key questions is, how do the nuclease domains become activated when the structural data suggests they are held far apart along the DNA. The data shows that nuclease domains do not need to interact directly, but instead can be activated by protein-protein contacts between distal parts of the protein. In addition, the data provides corroboratory evidence both for a 15 bp footprint for a translocating enzyme in a collision complex and for a role of ATP hydrolysis in the formation of a mobile collision complex necessary for formation of a double-strand break by ‘DNA shredding’ (Figure 1A).

**Figure 9.**
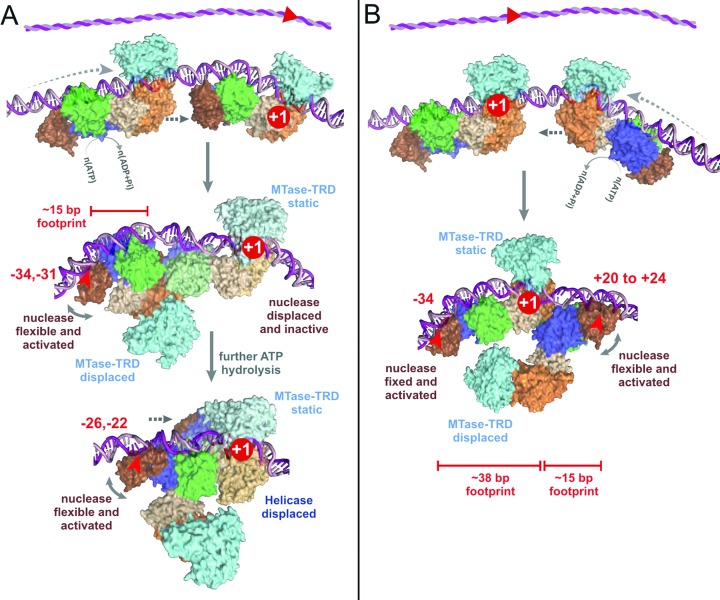
Models for DNA cleavage at a site following: (**A**) Rear-end collision; or (**B**) Head-on collision. Domains coloured as in Figure 1B,1C. The red +1 represents the methylated adenine at the site and marks the static enzyme in each complex. Here, we present the MTase-TRD displacement as occurring upon collision rather than during initiation of translocation. For rear-end collision (A), the static site-bound enzyme is *not* activated but provides a stepwise series of roadblocks as domains peel off the DNA (Figures 4 and 7). At each roadblock, the nuclease of the rearward translocating enzyme becomes activated by motor activity, sensed through its helicase domain (Figures 3 and 4). Once the static MTase-TRD releases its site, strain is released and further nuclease activity is curtailed. Thus the nicking events localise only at the site (Figure 3). For the head-on collision of two translocating enzymes (Figure 2B and C), motor-dependent activation of the nucleases is most likely via the helicase domains in both cases. However, for head-on collision between a motile enzyme and a static enzyme at a site (B), the interacting domains are different in each enzyme: the MTase-TRD of the static enzyme which is stably associated with the DNA; and the helicase domain of the translocated enzyme because of the displacement of the MTase-TRD. Nonetheless, both nucleases become activated (Figure 5).

The first set of results presented (Figures 3 and 4) showed that the single-strand DNA nicking that is produced by rear-end collision between a translocating enzyme and an enzyme bound at a site on HtT DNA (Figure 1A) (9,10), is due to activation of the upstream, motile enzyme. Although at first it might appear surprising that the LlaGI nuclease domain was not activated, it is consistent with the suggested model for the activated collision complexes that forms during head-on collision (Figure 2A and C) (8). In those complexes, protein-protein interaction occurs via the MTase-TRDs or, more likely (see below), via the helicase domains, and the nuclease domains are activated at a distance. In the case of rear-end collision (Figures 3 and 4), the protein-protein interaction will occur, initially at least, between the LlaGI nuclease and either the LlaBIII MTase-TRD or the LlaBIII helicase (Figure 9A). Since only LlaBIII becomes activated, it appears that the MTase-TRD and/or the helicase act as sensors that lead to activation of the upstream nuclease domain. This idea is corroborated below from the head-on collision data. Notably, the LlaGI nuclease is not activated by directly applied motor activity by an upstream translocating LlaBIII enzyme.

Each of the cleavage loci resulting from rear-end collision mapped in Figure 4C must represent positions where the LlaBIII nuclease was located in an activated state. Assuming no change in the relative domain order as shown in Figure 1B, and given that LlaGI would normally be expected to contact at least 30 bp upstream of its site (Figure 1B), the translocated LlaBIII must have displaced LlaGI domains off the DNA to allow placement of its nuclease domain in the region −34 to −22. One explanation of the cleavage loci observed is that they represent the products of multiple, unique collision complexes, where different LlaGI domains have been displaced fully or partially from the DNA. The idea that LlaGI domains can be separately displaced from the upstream side is corroborated by the footprinting time course data (Figure 7C). If LlaBIII covers ∼15 bp in the rear-end collision complex, as suggested in the Introduction and discussed further below, then the -34 and -31/-30 loci could represent displacement of the LlaGI nuclease domain and collision between the LlaBIII and LlaGI ATPase domains (Figure 9A, step 1). In agreement with this suggestion, collision of LlaBIII with LlaGIΔN which lacks the complete nuclease domain still produced the −34 and −31 products (lane 10 in Figure 4B). Subsequent displacement of the LlaGI ATPase would allow LlaBIII to move further downstream until blocked by the LlaGI coupler-MTase-TRD, locating the nuclease at −26/−22 (Figure 9A, step 2). Once the LlaGI coupler-MTase-TRD was displaced, further cleavage by LlaBIII was prevented, possibly by a resumption of translocation.

The next result presented (Figure 5) showed that head-on collision between a translocating enzyme and an enzyme at a site produced a discrete set of major cleavage loci on *both* strands. These nicks do not result in double-strand break formation because they are too far apart (55 bp apart at the shortest spacing) (11). The lack of further processing to produce a dsDNA break is most likely due to the fact that a LlaGI mutant was used which cannot hydrolyze ATP. Where both enzymes can hydrolyze ATP, collision at a site can produce dsDNA breaks (*e.g*., such collisions will be common for the short spacings in Figure 6). This data is therefore consistent with our suggestion that double-strand break formation requires a mobile collision complex and that the mobility requires ATP hydrolysis by *both* enzymes (8).

What is the collision interface that produced the activation during head-on collision at a site in Figure 5? Given that the 55 bp minimum spacing between the cleavage loci is greater than the 30 bp minimum seen normally (Figure 2C) (8), and because the location of the single LlaGI cleavage matches that seen for an isolated enzyme under ATP-independent conditions (Figure 1B), we suggest that the LlaGI MTase-TRD remained bound to its site in the collision complex and was not displaced by LlaBIII (Figure 9B). Therefore, the LlaGI domains that interacted with LlaBIII were most likely the MTase-TRD. If the LlaGI MTase-TRD was not displaced, then the +20 to +24 cleavages on the other strand are most consistent with a LlaBIII structure in which the LlaBIII MTase-TRD has swung off the DNA (Figure 9B); the cleavage sites are 14–18 bp away from the edge of the LlaGI site or, alternatively, 12–16 bp away from the main face of the LlaGI MTase-TRD (which encloses the DNA up to +8 as defined in Figure 1B and from the data in Figure 6). The LlaBIII nuclease domain could not be placed in the +20-+24 region if the MTase-TRD were still bound to the DNA. If this were the case we would have expected some cleavage events to have been observed in the region +40 to +60 (i.e. the complete LlaBIII footprint as predicted from Figure 1B). Thus the MTase-TRD domains must have been displaced *before* cleavage occurred. Either the MTase-TRD domains were displaced during initiation of translocation or they were displaced upon collision but before activation of cleavage. Despite apparently being in different structural configurations during head-on collision at a site, both LlaGI and LlaBIII were activated to cleave the DNA.

Our interpretation of the collision interface is consistent with LlaBIII having the previously suggested ∼15 bp footprint for a Type ISP enzyme in a collision complex. It is also consistent with a role for ATP hydrolysis in remodeling the MTase-TRD contacts. We note that the HtH collision complex studied here is somewhat artificial in that the LlaGI protein is an ATPase-deficient mutant. However, it is striking that the locations of the cleavage loci of both enzymes are consistent with predictions from previous observations using wild type proteins.

How do the nuclease domains become activated at–a-distance upon collision? Because cleavage requires at least one translocating Type ISP enzyme, we would suggest that ATP hydrolysis-dependent motor activity may play a role. The activating interactions could be due to the motor activity of the enzyme itself (e.g. the activation of LlaBIII when it collides with a static LlaGI – Figures 3–5) or due to the motor activity of a colliding enzyme (e.g. the activation of a static LlaGI ATPase mutant by translocating LlaBIII – Figure 5). The activation of LlaGI at a site by head-on collision but not by rear-end collision clearly illustrates that activation occurs due to interactions of C-terminal domains rather than direct contacts to the nuclease domain. It seems unlikely that a unique protein-protein interaction is required since activation can occur in both head-on and rear-end configurations. Moreover, from the head-on collision data (Figure 5) it would appear that both the Type ISP helicase *and* MTase/TRD can act as non-specific sensors of motor activity. It is notable that non-Type ISP protein roadblocks (*e.g.*, an EcoRI nuclease mutant or the Lac repressor) do not appear to result in strain activation of a colliding Type ISP enzyme (11). This is possibly because they were displaced whereas a partner Type ISP enzyme, in either orientation, is not. Questions that remain to be answered include: exactly what structural feature(s) act(s) as the strain sensor; how stable does the roadblock need to be; and, how is motor activity coupled to nuclease activation?

In summary, we found that while head-on collisions resulted in activation of the nuclease domains of both enzymes, rear-end collisions only resulted in nuclease activation of the downstream, translocated enzyme. We propose that the motor activity of the translocating enzyme(s) activates the nuclease domain(s) via distal interactions of the helicase or MTase domains. Direct collision of a motor with the nuclease domain instead resulted in its dissociation from the DNA. Exonuclease footprinting revealed distinct domain-DNA boundaries, and confirmed that domains can be separately displaced from the DNA. For the head-on collisions, the mapping of the cleavage loci is consistent with a ∼15 bp minimal footprint for a translocating species as predicted from our previous assays. Since we do not see evidence for significantly larger footprints, we suggest that the majority of the initial head-on collision events produce nicks that are ∼30 bp apart and that mobility of the complex increases this to give the wide range of broken ends observed. We also propose that mobility requires head-on collision, and ATPase activity of both enzymes in the complex. ATP hydrolysis is also required to remodel the DNA contacts of the Type ISP enzymes to produce the ∼15 bp footprint.

## Supplementary Material

SUPPLEMENTARY DATA
