# Supplementary material for: Mapping DNA cleavage by the Type ISP restriction-modification enzymes following long-range communication between DNA sites in different orientations
Source: Nucleic Acids Res. 2015 Oct 26;43(21):10430–43. doi: 10.1093/nar/gkv1129 (PMC4666363; doi:10.1093/nar/gkv1129)
Supplement: SUPPLEMENTARY DATA [file supp_43_21_10430__index.html]

Mapping DNA cleavage by the Type ISP restriction-modification enzymes following long-range communication between DNA sites in different orientations — SUPPLEMENTARY DATA 

# Mapping DNA cleavage by the Type ISP restriction-modification enzymes following long-range communication between DNA sites in different orientations

## SUPPLEMENTARY DATA

- SUPPLEMENTARY DATA
