# Supplementary material for: Mapping DNA cleavage by the Type ISP restriction-modification enzymes following long-range communication between DNA sites in different orientations
Source: Nucleic Acids Res. 2015 Oct 26;43(21):10430–43. doi: 10.1093/nar/gkv1129 (PMC4666363; doi:10.1093/nar/gkv1129)
Supplement: SUPPLEMENTARY DATA [file supp_gkv1129_nar-02629-h-2015-File011.pdf]

Supplementary Information to

The nuclease domains of Type ISP restriction enzymes are indirectly activated at-a-distance by strain produced by motor activity

Kara van Aelst[1], Kayarat Saikrishnan[2] and Mark D. Szczelkun[1]

[1]DNA-Protein Interactions Unit, School of Biochemistry, University of Bristol, Bristol, BS8 1TD, UK, and [2]Division of Biology, Indian Institute of Science Education and Research, Pune, 411008, India

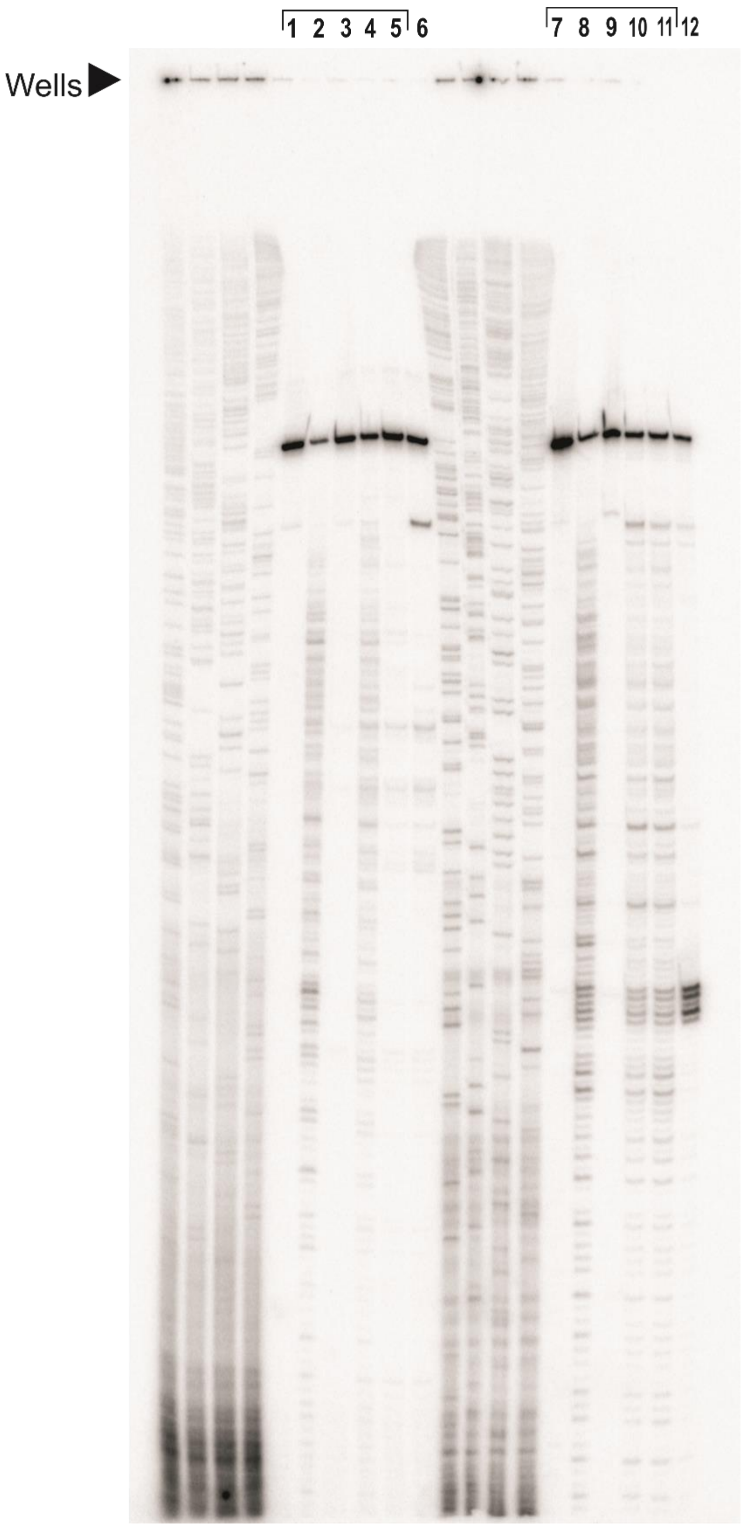

Supplementary Figure S1. Complete denaturing polyacrylamide gels used in Figure 5. Lanes 1-5 were deleted from the image for clarity. Lanes 6 (“bottom” strand) and 12 (“top” strand) are the samples lanes shown.
